# Differential Kinetics of Coagulation Factors and Natural Anticoagulants in Patients with Liver Cirrhosis: Potential Clinical Implications

**DOI:** 10.1371/journal.pone.0155337

**Published:** 2016-05-12

**Authors:** Michael Tischendorf, Wolfgang Miesbach, Umer Chattah, Zenab Chattah, Sebastian Maier, Christoph Welsch, Stefan Zeuzem, Christian M. Lange

**Affiliations:** 1 Medizinische Klinik 1, Klinikum der Johann Wolfgang Goethe-Universität Frankfurt, Theodor-Stern-Kai 7, Haus 11, 60590 Frankfurt, Germany; 2 Haemophilia Centre, Medical Clinic III / Institute of Transfusion Medicine, Klinikum der Johann Wolfgang Goethe-Universität Frankfurt, Theodor-Stern-Kai 7, 60590 Frankfurt, Germany; University of Navarra School of Medicine and Center for Applied Medical Research (CIMA), SPAIN

## Abstract

**Background:**

Advanced liver diseases are associated with profound alterations of the coagulation system increasing the risk not only of bleeding, but also of thromboembolic complications. A recent milestone study has shown that prophylactic anticoagulation in liver cirrhosis patients results in a reduced frequency of hepatic decompensation. Yet, INR measurement, one of the most widely applied tests to assess liver function, only inaccurately predicts the risk of hepatic decompensation related to alterations of the coagulation system. To assess the relationship between selected coagulation factors / natural anticoagulants with INR, MELD score, and hepatic decompensation, we performed the present pilot study. A total number of 92 patients with various stages of liver cirrhosis were included and prospectively followed for at least 6 months. We found that important natural anticoagulants, namely antithrombin and protein C, as well as factor XI (which may also serve as an anticoagulant) decreased earlier and by a larger magnitude than one would expect from classical coagulation test results. The correlation between these factors and INR was only moderate. Importantly, reduced plasma activities of natural anticoagulants but not INR or MELD score were independent predictors of hepatic encephalopathy (P = 0.013 and 0.003 for antithrombin and protein C, respectively).

**Conclusion:**

In patients with liver cirrhosis plasma activities of several natural anticoagulants are earlier and stronger affected than routine coagulation tests. Reduced activities of natural anticoagulants may be predictive for the development of hepatic encephalopathy.

## Introduction

Advanced liver diseases are associated with reduced plasma levels of most, though not all (e.g. factor VIII), coagulation factors [1]. Hence, advanced liver diseases are considered as a prototype of an acquired bleeding disorder, and especially gastrointestinal bleeding events contribute significantly to the mortality of patients with liver cirrhosis. Yet, there is increasing evidence that a decrease of natural anticoagulants such as antithrombin or protein C in parallel to coagulation factors may result in a rebalanced coagulation system in liver cirrhosis patients, and even an increased risk of thromboembolic events has been reported in patients with advanced liver diseases [1, 2].

Importantly, a number of studies have shown that neither the international normalized ratio (INR)–currently the most important single test to assess liver synthesis capacity–nor other classical coagulation tests perform well to predict the risk of gastrointestinal or procedure-related bleeding[1, 3, 4]. These clinical data are supported by results from experimental studies which have shown that classical coagulation tests do not adequately reflect the capacity of plasma from patients with liver cirrhosis to generate thrombin, the final factor in the coagulation cascade, which was partially explained by lacking inclusion of protein C and thrombomodulin in standard clinical coagulation tests [5, 6].

The profound alterations of the coagulation system in patients with advanced liver diseases may have important clinical implications beyond risk of bleeding and thromboembolic events. Animal and patient studies revealed a negative functional impact of alterations in the coagulation system on liver fibrosis progression, which can partially be explained by microthrombi within the hepatic microcirculation [7–9]. Even more important, a recent randomized milestone study has reported not only a reduced frequency of portal vein thrombosis but also an independent reduction of hepatic decompensations in patients with liver cirrhosis who were treated for 48 weeks with prophylactic enoxaparin [10].

To further elucidate the relationship between coagulation factors and natural anticoagulants in patients with liver cirrhosis and their possible relationship to hepatic decompensation, the present pilot study was performed.

## Patients and Methods

### Patients

Consecutive patients with liver cirrhosis who were admitted to the inpatient hepatology unit of the Goethe University Hospital Frankfurt from October 2014 to March 2015 were included and prospectively followed in the present pilot study. Inclusion criteria were proven liver cirrhosis by sonographic or histological methods, written informed consent to the study participation, and age ≥ 18 years. Exclusion criteria were presence of sepsis or systemic inflammatory response syndrome (SIRS), presence of acute bleeding, and intake of vitamin K antagonists or direct oral anticoagulants. Demographic and clinical characteristics, including age, sex, etiology of liver disease, complications of liver cirrhosis (hepatocellular carcinoma, hepatorenal syndrome, hepatic encephalopathy (HE), ascites, gastrointestinal bleeding), and results of laboratory analyses were extracted from clinical databases. HE was defined as present or absent whereas ascites was defined as absent/minimal, moderate, or massive, according to the judgement of the responsible physician. Since inclusion, patients are followed prospectively for above indicated complications of liver cirrhosis and death. The study protocol (protocol number 312/15)was approved by our local ethical committee (Ethical committee of the Faculty of Medicine, Goethe University Frankfurt, Germany).

### Selection and quantification of coagulation factors

In addition to international normalized ratio (INR), partial thromboplastin time (aPTT) and factor V, which are classical tests applied in hepatology to assess coagulation, a panel of coagulation and natural anticoagulation factors were selected for the present study. Protein C, protein S and antithrombin were selected as the most important natural anticoagulants of which reduced plasma levels in the general population clearly increases the risk of thromboembolic events. Furthermore, factor XI and XII were quantified for the present study, because these coagulation factors may represent important emerging targets for antithrombotic therapy [11, 12].

### Measurement of coagulation parameters

Coagulation tests, including PT, aPTT, and factor assays, were performed on an automated coagulation analyzer, ACL TOP (Instrumentation Laboratory, Lexington, MA, USA). PT was measured using the HemosIL RecombiPlasTin reagent (Instrumentation Laboratory,), and aPTT was measured using the SynthASil reagent (Instrumentation Laboratory). Fibrinogen was measured using the HemosIL Fibrinogen-C XL reagent (Instrumentation Laboratory) based on the Clauss method. Coagulation factors were tested using a PT-based clotting assay with the HemosIL RecombiPlasTin reagent (for FV) and an aPTT-based clotting assay using the SynthASil reagent (for FVIII, FXI, and FXII). Antithrombin and protein C activity were determined with chromogenic assays (HemosIL liquid antithrombin and HemosIL Protein C; Instrumentation Laboratory) and free protein S antigen with an immunoassay (HemosIL Free Protein S, Instrumentation Laboratory).

### Statistical analysis

Associations between dichotomic clinical endpoints (e. g. presence *vs*. absence of hepatic encephalopathy, moderate to high *vs*. none to minimal ascites) and above described clinical variables were assessed by logistic regression models. After univariate analyses, multivariate analyses were performed for significant associations. Multivariate models were obtained by backward selection, using a *P* value >0.10 for removal from the model. Only patients with complete data for the remaining covariates were included in multivariate analyses. Group differences were assessed by means of χ^2^ contingency tables or Wilcoxon-Mann-Whitney-U-tests, as appropriate.

## Results

### Baseline characteristics of included patients

A total of 92 patients were included in the present study and followed for at least 6 months. The MELD scores ranged from 6 to 40 including 12, 51, 24, and 5 patients with MELD scores <10, between 10 and <20, between 20 and < 30, and ≥30, respectively. Detailed patient characteristics are summarized in Table 1.

**Table 1:** Baseline characteristics of study population.

Characteristics	N = 92
Male gender, n (%)	68 (74)
Age (years), mean (IQR)	58 (51–66)
Sodium (mmol/L), mean (IQR)	133 (132–139)
Creatinine (mg/dL), mean (IQR)	1.4 (0.8–1.7)
Bilirubin (mg/dL), mean (IQR)	5.0 (1.3–4.6)
Albumin (g/dL), mean (IQR)	3.2 (2.7–3.8)
INR, mean (IQR)	1.5 (1.2–1.6)
MELD, mean (IQR)	17 (11–22)
Platelets (per nL), mean (IQR)	108 (67–137)
Ascites, n (%)	
none-minimal	30 (33)
moderate	23 (25)
massive	39 (42)
Hepatic encephalopathy, n (%)	
grade I-II	31 (34)
grade III-IV	7 (8)
Hepatorenal syndrome, n (%)	16 (17)
Death in 6 months, n (%)	29 (32)
Etiology of cirrhosis, n (%)	
viral	29 (32)
alcoholic	41 (45)
NASH	6 (7)
autoimmune	5 (5)
other/unknown	11 (12)

INR, international normalized ratio; IQR, interquartile range; MELD, Model of End Stage Liver Disease; NASH, non-alcoholic steatohepatitis.

### Dynamics of coagulation factors according to MELD score and INR

**Fig 1:**
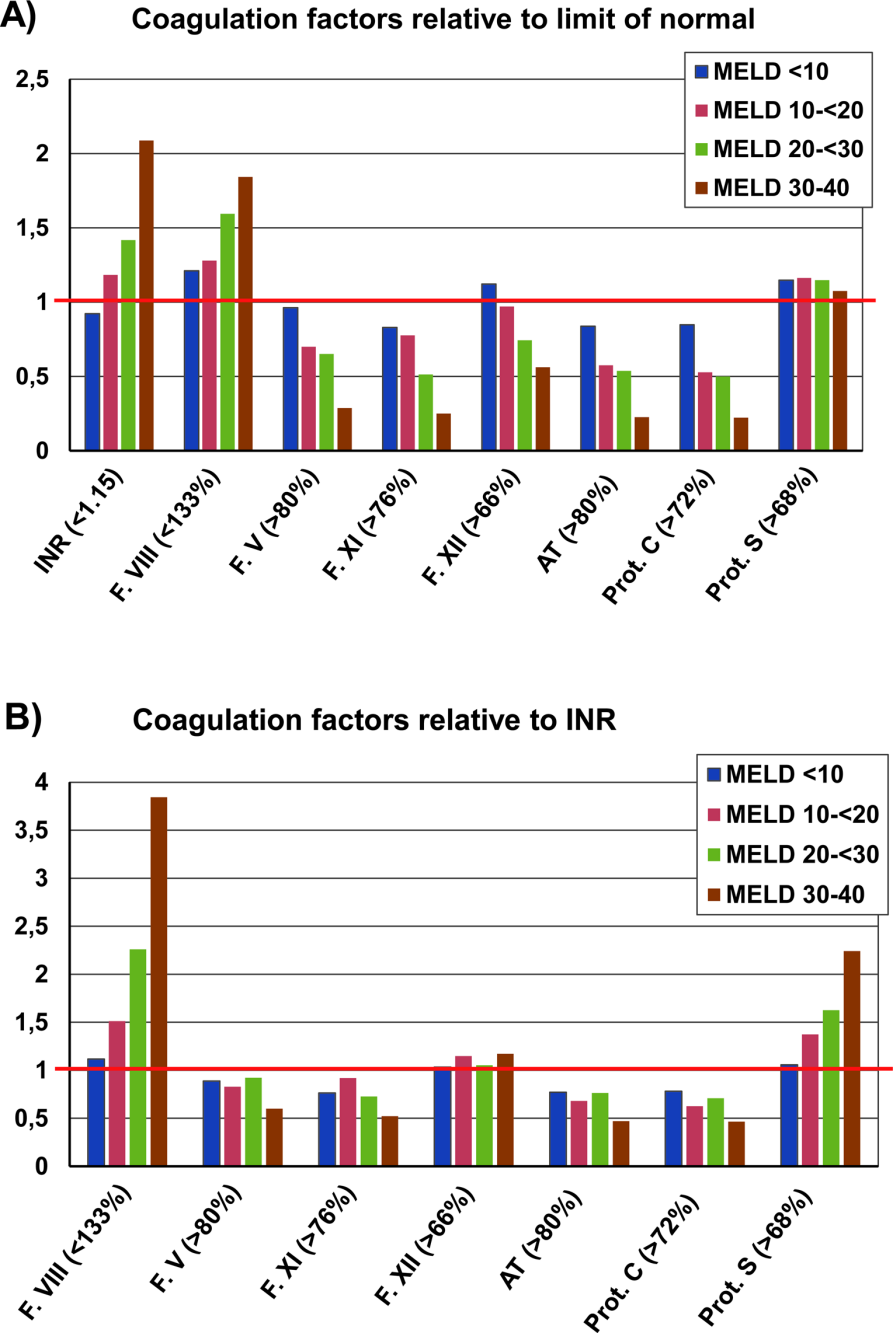
Kinetics of coagulation factors and natural anticoagulants according to MELD score and INR. Results are shown for patients stratified according to the indicated MELD scores. A) Mean INR and mean activities of coagulation factors / natural anticoagulants are shown as percentage of the upper / lower limit of normal. B) Mean activities of coagulation factors / natural anticoagulants are shown relative to the INR of patients.

To further characterize the coagulopathy of liver disease, plasma levels/activities of selected coagulation factors and natural anticoagulants were assessed according to the MELD score (Fig 1A). One can note that plasma levels of antithrombin and protein C, and–to a lesser extent of factor XI–are already significantly decreased in patients with low MELD scores (<10). In these patients, the mean INR was within the limit of normal. In patients with higher MELD scores, plasma levels of antithrombin, protein C, and factor XI decreased much stronger (more pronounced) than one would expect from the increase of INR (Fig 1A and 1B). In contrast, plasma levels of factor V decreased in parallel to deterioration of INR, with the exception of patients with end-stage liver disease (MELD score ≥30), Fig 1A and 1B. Furthermore, plasma levels of factor XII and–in particular–of protein S were only moderately or not at all affected by advanced liver disease. As expected, plasma levels of factor VIII were strongly increased in patients with liver cirrhosis [13]. Overall, these data show changes in coagulation factors and natural anticoagulants which develop only partially in parallel to INR and MELD score. This is further illustrated by the calculation of Pearson coefficients, which reveal a significant but imperfect correlation of natural anticoagulants with INR and MELD score (Table 2). In addition, multivariate testing showed that antithrombin (multivariate P = 0.01; beta = -0.03) and protein C (multivariate P = 0.03; beta = -0.03) independently (from INR) predict the MELD score, though this association is only of moderate strengths. Hence, measuring plasma levels/activities of these factors may add clinical information in addition to INR and MELD score quantification.

**Table 2:** Pearson correlation of coagulation factors / natural anticoagulants with INR and MELD score.

Factor	Pearson coefficient (*P* value)	
	INR	MELD Score
INR	n. a.	0.67 (P<0.00001)
Factor V	-0.54 (P<0.00001)	-0.04 (0.004)
Factor VIII	0.03 (0.7)	0.32 (0.005)
Factor XI	-0.67 (P<0.00001)	-0.70 (P<0.00001)
Factor XII	-0.51 (P<0.00001)	-0.42 (P = 0.0005)
Antithrombin	-0.60 (P<0.00001)	-0.44 (P = 0.00006)
Protein C	-0.69 (P<0.00001)	-0.41 (P = 0.0009)
Protein S	-0.06 (P = 0.6)	-0.06 (P = 0.6)
Fibrinogen	-0.52 (P<0.00001)	-0.25 (0.03)

INR, international normalized ratio; MELD, Model of End Stage Liver Disease.

### Decreased levels of anticoagulants may be associated with hepatic encephalopathy

Because prophylactic anticoagulation has been suggested to reduce the incidence of portal hypertension-related hepatic decompensation in patients with liver cirrhosis [10], we tested for associations between plasma levels of natural anticoagulants with the incidence of hepatic encephalopathy and the degree of ascites. Plasma levels/activities of antithrombin (P = 0.013) and protein C (0.003) were significantly associated with hepatic encephalopathy, whereas a statistical trend was observed for factor XI (P = 0.056). Table 3 shows results of a multivariable analyses, which revealed that the only independent predictors of hepatic encephalopathy in our cohort were antithrombin and hemoglobin concentration. Furthermore, statistical trends for associations between natural anticoagulants and the degree of ascites were observed (P = 0.077 for antithrombin, P = 0.68 for protein C), indicating a possible relationship between these variables.

**Table 3:** Multivariable analyses of predictors of hepatic encephalopathy.

	Univariate analysis		Multivariate analysis	
	OR (95% CI)	*P*	OR (95% CI)	*P*
Age (years, continuous)	0.99 (0.95–1.03)	0.9		
Male gender	0.56 (0.18–1.72)	0.3		
Creatinine (mg/dL, continuous)	0.77 (0.41–1.43)	0.4		
Plasma sodium (mmol/L, continuous)	1.00 (0.97–1.03)	0.9		
Bilirubin (mg/dL, continuous)	1.02 (0.94–1.10)	0.6		
Platelets (/nL, continuous)	0.99 (0.99–1.01)	0.5		
Hemoglobin (g/dL, continuous)	0.80 (0.67–0.99)	0.046	0.80 (0.66–1.00)	0.05
INR (continous)	1.84 (0.55–6.18)	0.3		
aPTT (seconds, continuous)	1.03 (0.97–1.09)	0.4		
Antithrombin (% of LLN, continuous)	0.97 (0.94–0.99)	0.031	0.97 (0.94–0.99)	0.037

LLN, lower limit of normal, INR, international normalized ration; aPTT, partial thromboplastin time.

## Discussion

The first major finding of the present study is that the change of plasma concentrations / activities of distinct coagulation factors and–in particular–of natural anticoagulants in patients with liver cirrhosis is inaccurately reflected by the INR or the MELD score. This applies in particular for antithrombin, protein C and factor XI, the activities of which appear to be earlier and stronger affected than one would expect from classical coagulation tests including INR. The second major finding of the present study is that decreased activities of natural anticoagulants, in particular of antithrombin and protein C, are independently associated with the occurrence of hepatic encephalopathy and with the MELD score.

Our findings may have prognostic implications as they further support the notion that routine coagulation tests do not accurately reflect the true status of the coagulation system in patients with liver cirrhosis. The INR, which is widely used in hepatology to quantify liver synthesis capacity and–as a variable of the MELD score–to allocate liver allografts, has been validated for patients who are treated with vitamin K antagonists. From a mechanistic point of view, it has already been stated that the INR should be re-standardized for the purpose of predicting mortality in patients with liver cirrhosis by the usage of plasma from patients with liver cirrhosis instead of using plasma from patients receiving vitamin K antagonists [1]. The rather moderate correlation of antithrombin, protein C and factor XI with the INR and the apparent decrease of these factors already in patients with normal INR may partially be explained by the inappropriate usage of the INR in cirrhosis patients. However, it appears likely that our data also reflect unequal kinetics of the changes of coagulation factors and natural anticoagulants during the course of liver cirrhosis, as for example the activity of protein S appears to be largely unaffected even in patients with end stage liver disease (Fig 1). An imbalance of pro- and anticoagulation factors, assessed by means of thrombin generations assays, has been reported in a previous study, especially in patients with Child-Pugh class C patients [5]. Hence, clinical testing of factors such as antithrombin or protein C may add clinical information to current routine diagnostic tests.

Our findings may also have important therapeutic implications. As described above, a recent milestone study by Villa *et al*. has shown that patients with liver cirrhosis may benefit from prophylactic anticoagulation therapy by reducing the risk of portal vein thrombosis and–strikingly–of hepatic decompensation events independently from the development of portal vein thrombosis [10]. The reduction of hepatic decompensation (with the notable exception of hepatorenal syndrome) by prophylactic anticoagulation was explained in this study by a reduced rate of bacterial intestinal translocation, which may be attributable to a reduction of intestinal and hepatic microthrombi generation. This attractive hypothesis may well explain the association between hepatic encephalopathy and reduced natural anticoagulants observed in our study. However, our study is not able to show any causal relationship between a decrease of natural anticoagulants and occurrence of hepatic encephalopathy. Therefore, further studies are required to replicate these associations and to further explore the mechanistic basis of our findings. In addition, future studies may be justified to assess whether anticoagulation reduces the incidence of hepatic encephalopathy, a complication of liver cirrhosis, which was only rarely reported in both the control group and the enoxaparin group of the Villa study.

Our study helps to better understand the differential kinetics of coagulation factors and natural anticoagulant during the course of liver cirrhosis. Such information may help to better guide future anticoagulation strategies in patients with liver cirrhosis. The pronounced reduction of protein C observed in our and other studies may caution the application of vitamin K antagonists in patients with liver cirrhosis, as these agents result in a further inhibition of protein C synthesis. In addition, the strong reduction of antithrombin may question the efficacy of heparins, which (at least partially) is dependent on sufficient amounts of antithrombin. Hence, novel direct oral anticoagulants (NOACs) may represent attractive agents for patients with liver cirrhosis, though concerns about their potential hepatotoxicity require attention and though detailed data on the course of the concentration of target molecules of NOACs (i.e. factor Xa and thrombin) during the progression of liver cirrhosis are lacking [14]. Theoretically, targeting factor XI or XII could be a suitable anticoagulation strategy, as recent clinical and experimental milestone studies have shown that inhibition of these factors leads to protection from thrombosis without increasing the risk of bleeding [11, 12].

Our study has several additional limitations. First of all, the number of enrolled patients in this pilot study was relatively small. Additional trials are necessary to confirm our findings. Furthermore, our study was not designed to and not sufficiently powered to assess patient survival. The lacking association between survival and coagulation factors / natural anticoagulants should therefore rather be considered as a preliminary result, in particular because we have observed a significant (though imperfect) association between natural anticoagulants and the MELD score. Finally, we were not able to perform tests which definitely assess the clotting capacity in our patient samples.

In conclusion, in patients with liver cirrhosis plasma activities of several natural anticoagulants are stronger affected as one would expect from INR measurements. There may be a functional link between the coagulation system and the development of hepatic encephalopathy.

## Abbreviations

AT: antithrombin
CI: confidence interval
INR: international normalized ratio
MELD: model of end stage liver disease
OR: odds ratio
PVT: portal vein thrombosis
aPTT: partial thromboplastin time
SIRS: systemic inflammatory response syndrome
